# Treatment of a rapidly expanding thoracoabdominal aortic aneurysm after endovascular repair of descending thoracic aortic aneurysm in an old patient

**DOI:** 10.1186/1471-2482-12-S1-S26

**Published:** 2012-11-15

**Authors:** Vito Mannacio, Michele Mottola, Danilo Ruggiero, Andrea D’Alessio, Giuseppina Gabriella Surace, Ettorino Di Tommaso, Bruno Amato, Gabriele Iannelli

**Affiliations:** 1 Department of Cardiac Surgery, University of Napoli Federico II, Napoli, Italy; 2 Department of General, Geriatric, Oncologic Surgery and Advanced Technologies, University “Federico II” of Naples, Italy

## Abstract

**Background:**

Aortic pathology progression and/or procedure related complications following endovascular repair should always be considered mostly in older patients. We herein describe a hybrid procedure for treatment of rapidly expanding thoracoabdominal aneurysm following endovascular treatment of a descending thoracic aortic aneurysm in an older patient.

**Case presentation:**

A 82-year-old man at 18 months after endovascular surgery for a contained rupture of descending thoracic aortic aneurysm revealed a type IV thoracoabdominal aneurysm with significant increase of the aortic diameters at superior mesenteric and renal artery levels. A hybrid approach consisting of preventive visceral vessel revascularization and endovascular repair of entire abdominal aorta was performed. Under general anaesthesia and by xyphopubic laparotomy, the infrarenal aneurysmatic aorta and common iliac arteries were replaced by a bifurcated woven prosthetic graf. From each of the prosthetic branches two reverse 14x7 mm bifurcated PTFE prosthetic grafts were anastomized to both renal arteries and to the celiac axis and superior mesenteric artery, respectively. Vessel ischemia was restricted to the time required for anastomosis. Three 10 cm Gore endovascular stent-grafts for a total length of 15 cm, were used. The overlapping of the stent-grafts was carried out from the bottom upwards, starting from the aorto-iliac prosthetic body up to the healthy segment of thoracic aorta, 40 mm from the previous stent-grafts.

The patient was discharged on the 9th postoperative day.

**Conclusion:**

This technique offers the advantage of a less invasive treatment, reducing the risk of paraplegia, visceral ischaemia and pulmonary complications, mostly in older patients.

## Introduction

The risk of thoracoabdominal expansion should be kept in mind after thoracic aortic repair [1].

Conventional surgical treatment still entails a substantial operative mortality [2].

To emphasize the possibility of an unforeseeable accelerating growth of aortic aneurysm and of an alternative treatment, we describe a case of a rapidly expanding thoracoabdominal aneurysm, treated by an hybrid procedure, after an emergent endovascular repair for a contained rupture of a descending thoracic aortic aneurysm.

## Presentation of the case

A 82-years-old man was admitted in June 2010 to the emergency care unit because of lypotimia and chest pain. After cardiologic evaluation, excluding coronary artery disease, the patient underwent spiral multislice computed tomography (CT-scan) showing a contained rupture of a descending thoracic aortic aneurysm, a maximum diameter of 63mm. 32 mm. distant from the origin of the left subclavian artery. A small aneurysm of entire abdominal aorta with a diameter of 42 mm and of 40 mm at the superior mesenteric and renal artery levels respectively was also found. As an emergency procedure, thoracic endovascular aortic repair (TEVAR), by positioning of three 42-42,46-42,44-40 mm stent-grafts (Talent, Medtronic Inc., Santa Rosa, USA), under local anaesthesia and light sedation, as previously described elsewhere [1],was successfully performed and the patient was discharged in good condition on the 5^th^ postoperative day. At six months follow-up, the patient did well and a CT-scan confirmed the complete exclusion of the thoracic aneurysm. The abdominal aortic aneurysm was unchanged. A yearly CT-scan monitoring was scheduled.

**Figure 1:**
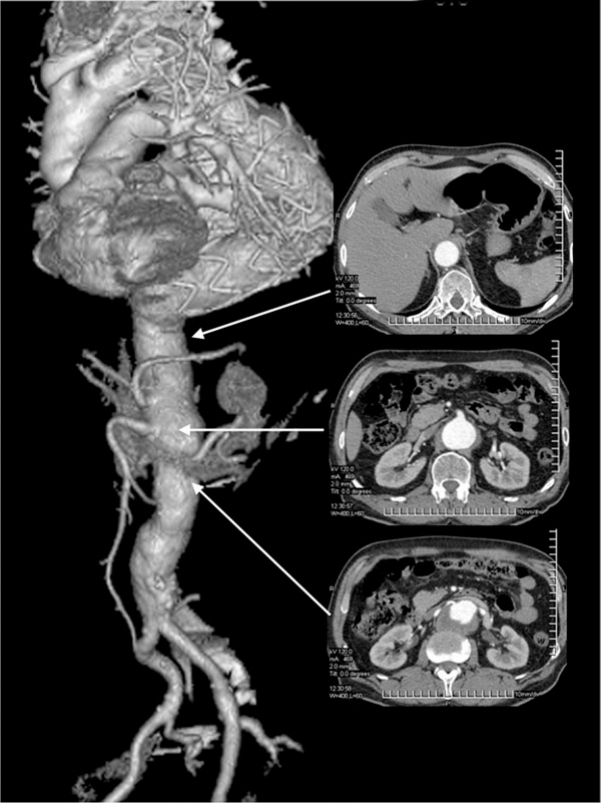
Angio CT scan of the thoracoabdominal aorta, performed 18 months after endovascular repair of thoracic aorta aneurysm: transverse reconstructions at the healthy segment of the descending thoracic aorta and at the superior mesenteric and renal artery levels.

**Figure 2:**
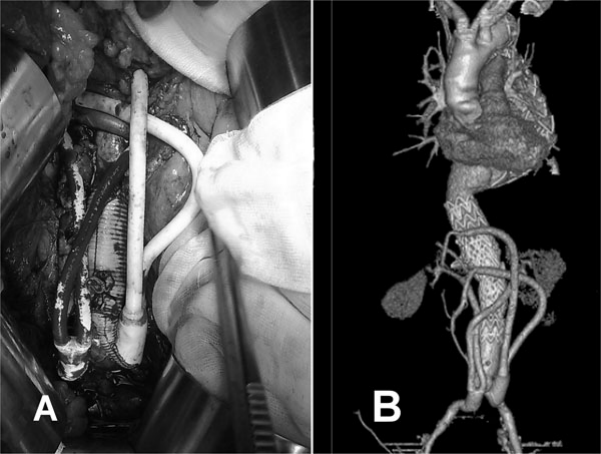
(A) Intraoperative view. (B) Early angio CT scan showing the implanted stent-grafts and the patency of all prosthetic bypass.

After 18 months from TEVAR, a CT-scan showed a type IV thoracoabdominal aortic aneurysm with significant increase of the aortic diameters up to 57.6 mm and 50.3 mm at the superior mesenteric and renal artery levels, respectively (Fig.1). A hybrid approach consisting of preventive visceral vessel revascularization and endovascular repair of the entire abdominal aorta was planned. Under general anaesthesia and by xyphopubic laparotomy the abdominal aorta, celiac axis, superior mesenteric artery, renal arteries and common iliac arteries were harvested. After heparinization (100 U/kg), the infrarenal aorta and common iliac arteries were replaced by a 20 x10 mm bifurcated woven prosthetic graft (Wovex, Bard Nordic, Sweden). From each 10 mm branches, two reverse 14x7 mm bifurcated PTFE prosthetic graft (WL Gore, Flagstaff, Ariz.) were anastomized to both renal arteries and to the celiac axis and superior mesenteric artery, respectively (Fig.2). Vessel ischemia was restricted to the anastomosis time.

Through the aorto-iliac prosthetic body, - three endovascular stent-grafts (TAG, W.L. Gore, Flagstaff, Ariz.), - 26-26,34-34,37-37 mm - were positioned from the aorto-iliac prosthetic body up to healthy segment of thoracic aorta 40 mm away from the previous stent-grafts. Intraprocedural angiography showed patency of the visceral vessels and good sealing of the stent-grafts. The patient was discharged on the 9^th^ postoperative day. A CT-scan performed at 6 months follow-up confirmed patency of all prosthetic bypass and re-implanted visceral vessels and complete exclusion of thoracoabdominal aneurysm, (fig.2).

## Discussion

TEVAR of descending aortic aneurysm is a widely accepted procedure, mostly in emergencies, as followed by us [3]. After endovascular repair the possibility of aortic pathology progression and/or procedure related complications should always be kept in mind.

Retrograde type A aortic dissection has been deemed a rare complication after endovascular stent graft placement for descending thoracic aortic diseases. The incidence is low (1.33 %) with an high mortality rate (42%) [4]. When the ascending aorta is involved open surgery for ascending aorta replacement was necessary. Conversely, if the dissection involved only the aortic arch, an endovascular treatment with transfemoral uncovered bare stent implantation is possible [5].

Although aneurismal growth may follow an indolent course, published reports (1-2) note that in older populations the descending and thoracoabdominal aorta grows at a rate of about 0.19 cm per year.

To avoid measurement error, we compared identical segments of the aorta in sequential studies measuring the aortic axis at the origin of the visceral vessels.

In this patient we observed, at the superior mesenteric artery level, a rapid and pathological overall growth of 1.53 cm (1.02 cm /year), requiring prompt treatment.

Despite the improvement of surgical and anaesthesiological techniques, open repair of a thoracoabdominal aneurysm still entails consistent risk of mortality and morbidity [2], mostly in older patients. As an alternative, a combined endovascular repair and retrograde visceral vessel revascularization has been considered [6].

To minimize the risk of paraplegia, the non-aneurysmatic aortic critical segment between T8 and T10 and the internal iliac arteries have been spared by endovascular and surgical approach, respectively. Furthermore, the reported technique of sequential visceral vessel revascularization reduces significantly life-threatening celiac and mesenteric vessel ischemia and acute renal failure. Finally, this hybrid approach, avoiding a wide thoracophrenolaparotomy, could decrease the incidence of pulmonary complications, the most common morbidity after open surgery, observed in more than 30% of patients even in excellent health [2,6-9]. Compared to conventional surgery, this technique, in older patients, offers the advantage of a less invasive treatment, reducing the risk of paraplegia, visceral ischaemia and pulmonary complications and could result on shorter length of hospital stay. Thus, this hybrid approach, despite requiring several prosthetic devices, could be a promising cost-effectiveness strategy.

## List of abbreviations used

ACT: activated clotting time; CT: computed tomography; PTFE: Polytetrafluoroethylene; TEVAR: thoracic endovascular aortic repair.

## Competing interests

The authors declare that they have no competing interests.

## Authors’ contributions

VM: conception and design, interpretation of data, given final approval of the version to be published; MM, DR, ADA, GGS, : acquisition of data, drafting the manuscript, given final approval of the version to be published; EDT: critical revision, interpretation of data, given final approval of the version to be published; AB, GI: conception and design, critical revision, given final approval of the version to be published.
